# When can the Child Speak for Herself? The Limits of Parental Consent in Data Protection Law for Health Research

**DOI:** 10.1093/medlaw/fwx052

**Published:** 2017-11-13

**Authors:** Mark J Taylor, Edward S Dove, Graeme Laurie, David Townend

**Affiliations:** 1 School of Law, University of Sheffield, UK; 2 School of Law, University of Edinburgh, UK; 3 Department of Health, Ethics & Society and CAPHRI Care and Public Health Research School, Maastricht University, The Netherlands

**Keywords:** Big Data, Children, Data protection, *Gillick*, Medical research, Parental consent

## Abstract

Draft regulatory guidance suggests that if the processing of a child’s personal data begins with the consent of a parent, then there is a need to find and defend an enduring consent through the child’s growing capacity and on to their maturity. We consider the implications for health research of the UK Information Commissioner’s Office’s (ICO) suggestion that the relevant test for maturity is the *Gillick* test, originally developed in the context of medical treatment. Noting the significance of the welfare principle to this test, we examine the implications for the responsibilities of a parent to act as proxy for their child. We argue, contrary to draft ICO guidance, that a data controller might legitimately continue to rely upon parental consent as a legal basis for processing after a child is old enough to provide her own consent. Nevertheless, we conclude that data controllers should develop strategies to seek fresh consent from children as soon as practicable after the data controller has reason to believe they are mature enough to consent independently. Techniques for effective communication, recommended to address challenges associated with Big Data analytics, might have a role here in addressing the dynamic relationship between data subject and processing. Ultimately, we suggest that fair and lawful processing of a child’s data will be dependent upon data controllers taking seriously the truism that consent is ongoing, rather than a one-time event: the core associated responsibility is to continue to communicate with a data subject regarding the processing of personal data.

## I. INTRODUCTION

Does consent obtained from a parent for use of a child’s personal data for health research ‘lapse’ when the child is old enough to provide her own consent? If so, when does that lapse occur, how is it to be determined, and with what consequences? Somewhat surprisingly, despite the increasing use of personal data from adults and children alike for health research, hitherto these questions have not been addressed in the literature or as a matter of data protection law.

In this article, we explore the implications of adopting ‘*Gillick* competence’—drawn from healthcare law—as the relevant test of sufficient maturity in the data protection law context.^1^ We adopt the familiar medico-legal language of the ‘mature minor’. This is intended to capture the moment when a child demonstrates sufficient autonomy—in a given context—to make a consent decision on her own behalf. Our discussion takes a UK data protection perspective, both in the current context (under the Data Protection Act 1998 or ‘DPA 1998’, which transposes the 1995 EU Data Protection Directive 95/46/EC) and also the future position (under the EU General Data Protection Regulation 2016/679 or ‘GDPR’, enforceable from May 25 2018, and national legislation put forward through the Data Protection Bill 2017). We focus our thoughts on the implications for health research of recognising a mature minor to be able to exercise the rights of a data subject on her own behalf. Particularly, we centre our analysis on the implications for the kind of longitudinal research that begins with a parental consent and continues for many years. While we choose this as our focal point, we note that the regulator’s suggestion that the *Gillick* test has something to offer data protection law is not limited to [the context of] healthcare, research, or health research. The points we make may, therefore, have broader application. At the end of the article, we reflect upon additional suggestions by the regulator that one should consider alternatives to consent, where they are available under data protection law.

The article adopts the following structure: first, we consider the rights of mature minors in the healthcare context. Here we consider the *Gillick* test developed in relation to children below the age of 16 and the operation of the Family Law Reform Act (FLRA) 1969. We explore the complexities of the tests as they change with the growing autonomy of a child, the contexts in which autonomy will or will not be recognised, and we lay the groundwork for exploring the implications of this for recognising children to be able to exercise the rights of data subjects in their own right. Next, we consider the age of legal capacity in the context of data protection and the implications of mapping the *Gillick* test across from the healthcare context. This raises crucial questions. For example, if *Gillick* is applied in data protection, then how can the child’s emerging competence be accommodated and how are the parents’ continuing responsibilities as the child’s proxy to be exercised? From the point an individual is judged first able to exercise any right of a data subject, can she *exclusively* exercise *all* the rights of a data subject or does her ability to exercise rights gradually unfold alongside a parallel (albeit diminishing) duty of a parent? What is the significance of the fact that, in both healthcare and data protection domains, existing guidance states that the core legal principle to be respected is the *best interest of the child*? How do on-going best interest considerations intersect in this context with an assessment of (decision-specific) maturity pre- or post-age 16? What are the responsibilities of a parent in such a shifting scenario? Finally, we consider how the answers to these questions relate to the UK Information Commissioner’s Office’s (ICO) recommendations regarding best practice in the context of Big Data analytics, an area of increasing application and importance in data protection law. Will consent even continue to be relevant from a data protection perspective for the processing of children’s data, when data are to be used to support health research? We conclude by considering the prospect of a move away from consent as the relevant legal basis for data processing.

These are significant issues to explore, not least because draft guidance on the forthcoming General Data Protection Regulation (GDPR) issued by the ICO in March 2017 states: ‘Parental consent will always expire when the child reaches the age at which they can consent for themselves. You [as data controller] need therefore to review and refresh children’s consent at appropriate milestones.’^2^ This guidance presupposes clear answers to the fundamental questions introduced above. Moreover, irrespective of the issue of *when* maturity is achieved, it remains unclear whether the ICO’s intention is to suggest that the validity of any consent provided by a parent lapses *as soon as* a data subject is able to exercise rights on her own behalf. If so, this would seem to be inconsistent with other guidance offered by data protection authorities as well as the operation of the *Gillick* test itself in the healthcare context, which recognises autonomy in ways that are themselves highly *context-specific*. We argue that a straightforward legal transplant of the *Gillick* test is problematic and presents considerable practical and ethical difficulties. This article, then, seeks to provide clarity for academics, data controllers, and regulators alike.

While it is possible for there to be different kinds of legal representative, in this article we focus on the possibilities surrounding parental consent. Our overarching aim is to demonstrate the critical importance of establishing clearly both when an individual is entitled to exercise the substantive rights of a data subject for herself, and how that entitlement procedurally meshes with the parental exercise of a data subject’s rights on her behalf. Clarity is a pre-requisite for an understanding of the legal and ethical responsibilities of a data controller processing personal data for health research purposes. It is also a pre-requisite for explanation to a data subject, and her parents, of her right to speak for herself.

## II. THE RIGHTS OF MATURE MINORS

In the UK, the age of full majority is 18.^3^ However, an individual is entitled to exercise certain legal rights while still a minor. An example is provided in the context of healthcare and treatment. Section 8(1) of the FLRA 1969 provides that in England and Wales,

[t]he consent of a minor who has attained the age of sixteen years to any surgical, medical or dental treatment which, in the absence of consent, would constitute a trespass to his person, shall be as effective as it would be if he were of full age; and where a minor has by virtue of this section given an effective consent to any treatment it shall not be necessary to obtain any consent for it from his parent or guardian.

Further, the courts have established that a valid consent may be given to healthcare by a child below the age of 16 *if* they are competent to make the occurrent decision. Competence here is assessed according to what is known as the test for *Gillick* competence (and what are called the *Fraser* Guidelines). We proceed to elaborate on *Gillick* competence and its relationship with the FLRA below.

### A. The *Gillick* Test in Healthcare

The case of *Gillick v West Norfolk & Wisbech Area Health Authority*^4^ established that where a person under the age of 16 has reached sufficient maturity to understand the nature and consequences of a proposed intervention, and it is in their best interests to do so, then they can provide a valid legal consent on their own behalf. This is a functional, rather than status-based, test of competence. In other words, a finding of competence is decision specific; competence is assessed at every decision point as it relates to the healthcare intervention in question. *Gillick* was concerned with contraceptive advice, and, in this context, Lord Fraser concluded that while a doctor should always seek to persuade a child to tell a parent if she is seeking contraceptive advice,

… there may well be cases, and I think there will be some cases, where the girl refuses either to tell the parents herself or to permit the doctor to do so and in such cases, the doctor will, in my opinion, be justified in proceeding without the parents' consent or even knowledge provided he is satisfied on the following matters: (1) that the girl (although under 16 years of age) will understand his advice; (2) that he cannot persuade her to inform her parents or to allow him to inform the parents that she is seeking contraceptive advice; (3) that she is very likely to begin or to continue having sexual intercourse with or without contraceptive treatment; (4) that unless she receives contraceptive advice or treatment her physical or mental health or both are likely to suffer; (5) that her best interests require him to give her contraceptive advice, treatment or both without the parental consent.^5^

If a child is not *Gillick* competent and is deemed to lack the capacity to consent to a healthcare intervention, then any valid and lawful consent must be given on her behalf by someone with parental (or other legal) responsibility or by the court. Here, it is worth noting that beyond the child’s capacity to understand, in *Gillick* the court was keen to ensure continued parental involvement so far as was consistent with the child’s best interests. In all cases, the proxy decision maker has a duty to keep the child’s best interests at the heart of any decision, and the child should be involved in the decision-making process as far as possible (eg by providing her assent).^6^ Although developed in the context of access to contraceptive services, *Gillick* has been extended to all aspects of medical treatment and care. In Scotland, its spirit is embodied in statute.^7^ This is so in two respects: first, the Scottish provisions are restricted to recognising a right to consent only to medical and dental treatment (and does not extend to other interventions such as health research), and secondly, the putative ‘right’ is only to consent, not to refuse. This reflects developments subsequent to *Gillick*, and to which we now turn.

The English and Welsh courts have established, through cases such as *Re R*^8^ and *Re W*,^9^ that it is possible for an individual refusal of treatment to be overridden where the court considers that to be in the child’s best interests. Thus, parental and court consent *in the minor’s best interests* continues to be valid even where a child is competent to make a particular decision but purports to offer a refusal.^10^ The distinction between consent and the ‘right to refuse’ medical care and treatment has been the subject of extended academic discussion.^11^ The significance of the distinction for our discussion is 3-fold: first, for a child under 16, any finding of competence will only relate to *a* decision. This is a narrow decision-specific competence, as established above; thus, if *Gillick* competence is to be extended to the data protection context, the implications of this need to be considered. Secondly, and relatedly, the pre-16 child’s competence to make a consent decision under the *Gillick* test exists in parallel with duties of others to act as proxy for them (with his or her best interests as an overriding consideration). This is very different from any suggestion that a child, essentially, attains majority (ie an absolute right to choose for herself) prior to the age of 18.^12^ Thirdly, given that *Gillick* only applies to children below age 16, we must also ask about the situation from age 16–18 and it is here that we return to the FLRA 1969.

It will be recalled that Section 8(1) of the 1969 Act, above, provides that a child between age 16 and 18 can consent to her own surgical, medical, or dental treatment. Moreover, Section 8(3) of the Act provides: ‘[n]othing in this section shall be construed as making ineffective any consent which would have been effective if this section had not been enacted.’ What, then, is the relationship between the common law (*Gillick*) minor and the statutory minor in terms of rights, responsibilities, and duties? An initial observation is obvious: while the competence of a pre-16-year-old must always be demonstrated and is only valid for each decision in which it is assessed, the threshold of 16 brings with it a presumption of competence with respect to the treatments specified in the statute. It follows, however, that this is not a general legal competence for other spheres of life, including health research: age 16–18 remains a liminal space. Less obvious, for want of any specific mention in Section 8 of the 1969 Act, are the tricky questions of (i) the role of best interests, and (ii) the position with respect to refusals. These have, however, been dealt with by the courts, notably by Lord Donaldson in *Re W* above. In contemplating the mischief that the 1969 Act was designed to address, Lord Donaldson revisited the report of the Latey Committee whose recommendations formed the basis of the Act.^13^ Despite the fact that all professional bodies^14^ giving evidence recommended that a consent between age 16 and 18 should also include a refusal, the Committee—and subsequently the Act—only made mention of consent, thereby rejecting such evidence. As Lord Donaldson himself remarked: ‘I am quite unable to accept that Parliament in adopting somewhat more prolix language was intending to achieve a result which differed from that recommended by the committee.’^15^ He then added: ‘[w]hat I am clear about is that Parliament has not conferred such autonomy on a 16- to 18-year-old child by virtue of section 8 of the Act of 1969, and that the common law, as interpreted by the House of Lords in Gillick’s case does not do so either.’^16^ This was subsequently confirmed in *Re E*, in which the refusal of a blood transfusion by a child of age 15 and three-quarters was overridden on the basis of his best interests.^17^ More significantly, it was not until he reached age 18 that his refusal was respected and he died as a result. This therefore also confirms that best interest paramountcy endures in the period between age 16 and 18, despite no mention in the 1969 Act.

The principle that underpins both the common law and the statutory regime was probably best captured by Lord Denning MR, shortly after the passing the 1969 Act:

The common law can, and should, keep pace with the times. It should declare, in conformity with the recent Report of the Committee on the Age of Majority … that the legal right of a parent to the custody of a child ends at the 18th birthday: and even up till then, it is a dwindling right which the courts will hesitate to enforce against the wishes of the child, and the more so the older he is. It starts with a right of control and ends with little more than advice.^18^

Before proceeding, we would add a few caveats. It should not be thought that all refusals are made equal; that is, the leading cases in this field all involved life-threatening situations and there are many other situations in which mature minors seek control over their lives that pose no threats to their lives. To override such decisions in the name of best interests becomes an increasingly untenable response over time. When we therefore contemplate decisions about control of one’s own data in a non-treatment context, we must be cautious about how far this line of precedent assists us. We return to the significance of the threshold of 16–18 in Section III, after turning to the specific content of data protection itself.

### B. Who is Entitled to Exercise the Data Subject Rights of a Mature Minor?

In the realm of data protection, regulatory guidance from both the regulator (ICO) and the Article 29 Data Protection Working Party states that it is possible for a sufficiently mature minor to be able to exercise, and not just hold inchoately, the rights of a data subject. For example, the Article 29 Data Protection Working Party^19^ offers guidance on the issue in their Opinion on the protection of children’s personal data.^20^ Consider the following quote:

If the processing of a child’s data began with the consent of their legal representative, the child concerned may, on attaining majority, revoke the consent. But if he wishes the processing to continue, it seems that the data subject need give explicit consent wherever this is required.

For example, if a legal representative has given explicit consent to the inclusion of his child (the data subject) in a clinical trial, then upon attaining capacity, the controller must make sure he still has a valid basis to process the personal data of the data subject. He must in particular consider obtaining the explicit consent of the data subject himself in order for the trial to continue, because sensitive data are involved.^21^

These paragraphs encompass two distinct concepts. The idea of ‘attaining majority’ is a reference to a bright line, such as the age of 18, while ‘attaining capacity’ might refer to a factual state of affairs when a child is able to understand and consent for herself, irrespective of her age and when the law must recognise her autonomy in respect of the particular decision in question. Indeed, this latter possibility is entirely envisioned by the Article 29 Working Party:

Since the child is a person who is still developing, the exercise of their rights – including those relating to data protection – must adapt to their level of physical and psychological development. Not only are children in the process of developing, but they have a right to this development.^22^

Regulatory guidance on data protection law in the UK also suggests that it is possible for a sufficiently mature minor to exercise the rights of a data subject.^23^ The ICO’s draft guidance on the GDPR states that when considering if a data subject can consent for herself, the general rule (outside offering of information society services, discussed below) is:

… you [as data controller] should consider whether the individual child has the competence to understand and consent for themselves (the ‘Gillick competence test’). In practice, you may still need to consider age-verification measures as part of this assessment, and take steps to verify parental consent for children without competence to consent for themselves.^24^

However, as noted above, the ‘*Gillick* competence test’ was developed in the context of healthcare and treatment.^25^ In that context the law recognises an individual’s capacity to make an occurrent decision on consent below the age of full majority at 18 years old: competence is functionally assessed in relation to a specific decision and is neither an abstract nor a generic assessment. What is more, a child’s competence to make a decision does not proscribe the possibility of a parallel, and potentially overriding, parental consent, when the child purports to refuse.

The ICO’s draft guidance thus raises a matrix of interesting questions related to when, and to what extent, a child can speak for herself, and the changing nature of the legal ability and duty of a parent to speak for her. In what follows, we tease out and address these questions.

### C. When Can a Child Speak for Herself in the Data Protection Context?

The rights of a data subject exist (at least) from birth.^26^ The relevant question here is: when are data subjects able to exercise those rights, who is able to *exercise* those rights along the trajectory of the data subject’s life, how should people other than the data subject do that, and what are the on-going implications of them doing so? More particularly, we must ask: from the point an individual is judged first able to exercise the rights of a data subject, can she *exclusively* exercise *all* the rights of a data subject (and *must* she be able to exercise all the rights to be able to exercise part), or does her ability to exercise rights gradually unfold alongside a diminishing but parallel duty of a legal representative to exercise those rights as a proxy?

The Article 29 Data Protection Working Party suggests that:

Where consent is concerned, the solution [of determining the exercising of data subject rights] can progress from mere consultation of the child, to a parallel consent of the child and the legal representative, and even to the sole consent of the child if he or she is already mature.^27^

Unfortunately, this is ambiguous guidance. It suggests that a mature minor can provide a sole consent *and* that, before it is possible for consent to be given solely by a child, the child and the parent may consent *in parallel*. Yet a child could not give a valid *parallel* consent unless she was deemed sufficiently mature to do so, and how is the state of maturity required for a parallel consent to be contrasted with the circumstance when she is sufficiently mature to provide a ‘sole consent’? Is reference to ‘the sole consent of the child’ intended to imply that, when a child can provide a sole consent, then a parent may no longer consent on her behalf? While perhaps consistent with the notion of emerging autonomy, this last suggestion is not consistent with parents continuing to have parental responsibilities in relation to a child’s welfare. Welfare considerations remain paramount here as in the healthcare context: guidance from the Article 29 Data Protection Working Party has stated that, within a data protection context, ‘The core legal principle is that of the best interest of the child.’^28^ As in the context of healthcare, parents continue to have responsibility—where necessary, expressed through parental consent—to act in a child’s best interest.^29^

Recalling the interplay between pre-16 common law (*Gillick*) and the statutory 16–18 regime outlined above—which fixes age 16 as the point at which a presumption of competence emerges—we argue that it would be ethically preferable and legally defensible for data controllers to envision (or, depending on their degree of involvement, ascertain) the emerging ability of an individual to exercise first the rights of a data subject *jointly* (rather than in parallel) with a parent—as it will be through dialogue that the child’s best interests are ascertained—and then a subsequent point in time at which a child is sufficiently mature to exercise her data subject rights *independently* (rather than solely) for particular isolated decisions, and then increasingly areas of decision-making on the way to sole decision-making.^30^ The age of 16 would represent a *prima facie* threshold for independent decision-making, while *Gillick* competence below age 16 might also demonstrate independence in a given context. At no point, however, does the residual role for best interests fall away. However, the ICO draft guidance above would suggest that—if the child is *Gillick* competent—then the parents are no longer entitled under the law to exercise the rights of the data subject on her behalf. This can only sensibly follow application of the *Gillick* test if ability to consent also implies an ability to refuse, but that is *not* what is implied by a simple import of the *Gillick* test from the healthcare context.

If competence to refuse is limited in healthcare to those cases where refusal is recognised to be in the best interests of the child, then we might expect similar limitations on the exercise of autonomy in the context of data protection. Put otherwise, at the point of relevant maturity, a child can exercise *all* the rights of a data subject *conditional* on that being consistent with her best interests. Thus, if a refusal to permit processing is not consistent with her best interests, then her ability to refuse is limited and parental responsibility to provide consent to data processing on her behalf persists. The alternative is to deny the continuing availability of a parental consent to processing in the child’s best interests. This said, and as indicated above, it is worth recognising that a refusal to permit personal data processing may be expected to interfere with her best interests less regularly than refusal to receive life-saving medical treatment. The overriding point here is that parents may continue to provide a valid consent even where a child satisfies the test of *Gillick* competence in relation to a particular decision.

This informs how we argue the Article 29 Working Party guidance is best interpreted. The question of who is entitled to exercise the data subject rights of a mature minor is best answered thus: parents are entitled, and required, to exercise the rights as proxy in *consultation* with the child or when engaging the child *jointly* in a decision-making process. The child is *independently* entitled when demonstrating sufficient maturity in the decision-making process to others charged with protecting her best interests, and conditional only upon her decision being in her best interests. The ability of a child to provide an independent consent does not proscribe her parents exercising a parallel right to provide a consent on her behalf where this is consistent with her best interests until the age of 18.

The guidance itself does not address the resolution of any potential conflict scenario, for example: if a parent consents to processing and a child purports to refuse. Our reading and interpretation of the guidance suggests resolution of such conflicts consistent with the operation of the *Gillick* test in healthcare law. The *Gillick* test has been preferred by the ICO, and the priority of best interests has been recognised by the Article 29 Working Party. However, neither the Article 29 Working Party nor the ICO draft guidance acknowledges explicitly the decision-specific nature of a competence assessment. If *Gillick* operates in the context of data protection, as it does in the context of healthcare, then we would expect any assessment of competence to be valid *only* relative to a particular exercise of data subject rights. This is an important qualification and supports the view, as we will express later, that in the absence of a specific reason to think otherwise, then there must be a presumption that parental consent remains valid until age 16, and as a minimum the assessment and implementation of best interests ought to occur jointly between the child and her parents throughout childhood.

As noted above, the Article 29 Working Party has recognised that ‘not only are children in the process of developing, but they have a right to this development’.^31^ Guidance on the validity of consent must be read in a way that recognises parents to have responsibilities to do more than simply act as proxy for their child. There is a duty to support the emerging competent child to a position where she can exercise the rights of a data subject herself.^32^ Might this be complemented by a duty towards the emerging competent child that can be discharged by the data controller or public authority? There are a number of ways that such a duty might be met. For example, might a controller provide parents with a form (or online contact details, etc.) to give to the child when the parents have reason to believe the child understands? Might a public body, such as the relevant data protection Supervisory Authority or a health research regulatory authority, create a central register of participation in research, so the mature or maturing minor knows where to go to find out the projects in which she is involved? Answering these questions sits outside the frame of this article but, we suggest, they are important for data controllers and regulators to consider. As we argue below, it is likely to be in the data controllers’ own interests to ensure that a child is effectively engaged in decisions relating to the processing of her data as early as possible.

#### 1. Children in Scotland

The Data Protection Bill 2017 makes special provision for children in Scotland. Clause 187 provides that where ‘a question falls to be determined in Scotland as to the legal capacity of a person aged under 16 to

- exercise a right conferred by the data protection legislation, or
- give consent for the purposes of the data protection legislation,’^33^

then, the ‘person is to be taken to have that capacity where the person has a general understanding of what it means to exercise the right or give such consent’.^34^ A person ‘… aged 12 or over is to be presumed to be of sufficient age and maturity to have such understanding, unless the contrary is shown’.^35^

The implication of our argument thus far is that there will be little variation in practice between Scotland, England, and Wales. One potential variation that does warrant consideration is the difference between ‘full understanding’ and ‘general understanding’. In the context of healthcare, in relation to *Gillick* competence, the courts have stated

… what is involved is not merely an ability to understand the nature of the proposed treatment - in this case compulsory medication - but a full understanding and appreciation of the consequences both of the treatment in terms of intended and possible side effects and, equally important, the anticipated consequences of a failure to treat.^36^

A ‘full understanding’ would seem to clearly exceed the ‘general understanding’ required of Scottish children. We would suggest that the context of data protection be relied upon to distinguish the approach taken by English courts in an application of the *Gillick* test in the healthcare context. It would seem most appropriate to expect, and to support, a ‘general understanding’ in relation to children on either side of the border.

### D. The Lawfulness of Continued Data Processing When a Child under Age 16 Attains *Gillick* Competence, and the Role of Best Interests

Underlying this discussion is the key issue of whether it is lawful for data processing to continue when a child under the age of 16 attains *Gillick* competence in relation to a decision, when consent was originally provided by a parent. Let us recall that the draft ICO guidance states that parental consent ‘will always expire when the child reaches the age at which they can consent for themselves’. If a child is able—in principle, but not necessarily in practice—to provide independent consent when *Gillick* competent, then does this draft guidance imply that any consent provided previously by a parent automatically expires? If so, this would present considerable practical and ethical difficulties. Practically speaking, a data controller may have no means of determining when an individual achieves competence beyond the initial contact with the parent and child and parental consent. This difficulty is compounded when one recognises that a finding of *Gillick* competence relates only to a particular decision. Ethically speaking, even if assessed in relation to a specific decision, eg on participation, then unless welfare considerations are brought into play, an assessment of competence could result in data processing stopping immediately even though it could be in the best interest of a child for it to continue (eg enrolment in a long-term paediatric clinical trial). This might undermine the medical purposes of processing and may not be desirable from the perspective of the controller (eg a research institution) or, more importantly, the data subject.

As we have seen, best interest is a crucial consideration when parental consent might expire—along with data processing. We have noted that Guidance from the Article 29 Data Protection Working Party recognises the best interests of the child to be the ‘core legal principle’.^37^ This suggests that provided processing is in the best interest of a child, then a previously provided parental consent should continue to be understood to be a lawful basis for processing, even though a child *might* have attained *Gillick* competence at a point thereafter for a (narrow) decision on that processing. Moreover, we note that the United Nations Committee on the Rights of the Children views best interests as a threefold concept involving (i) a substantive right, (ii) fundamental interpretative legal principle, and (iii) a rule of procedure. While it is ‘complex’, ‘flexible’, and ‘adaptable’, it is always the primary consideration.^38^ Thus, we would argue that where *Gillick* competence is the relevant test for determining whether a minor may exercise data subject rights, this entails that proxy consent by the parent will remain a valid option and continuing legal basis *if* the processing is in the child’s best interest. It is important to recall that as a general principle of law, parents can *only* lawfully exercise their rights and responsibilities within the parameters of their children’s welfare.^39^ Therefore, it may be assumed by a data controller to be a rebuttable presumption that to continue processing on the basis of an enduring parental consent will be in the best interests of the child.

This is the case even where a controller suspects that an individual is perhaps *likely* now to meet the test of *Gillick* competence, eg they have reached 14 or 15 years of age, *if* timely re-contact for the purposes of seeking fresh explicit consent from the mature minor is not practicable. There is an argument that it would not be in the data subject’s own best interest for processing to stop until or unless that further express consent can be obtained. Here it may be significant that the example given by the Article 29 Data Protection Working Party in its Opinion does not suggest that consent *must* be obtained. They state instead that ‘it seems that the data subject need give explicit consent *wherever this is required*’ (emphasis added).^40^ In the example given by the Working Party, the data controller is required to *consider*, when a legal representative has given explicit consent to the inclusion of his child (the data subject), whether ‘he still has a valid basis’ to process sensitive personal data upon the child attaining capacity.

In terms of a data controller’s responsibilities, at least three issues arise from this. First, does the data controller have reason to believe he does not have an enduring consent from the parents that provides a lawful basis for on-going processing? Secondly, does the data controller have any reason to believe that the continuing processing of the data would not be in the best interests of the child? Thirdly, does the data controller have a practicable opportunity to seek consent directly from a child where they have reason to believe that the child has achieved sufficient maturity to make a decision for herself (for example, communication from the child directly in relation to the processing operation)? If the answer to each of these questions is ‘no’, then it would appear appropriate to recognise the parental consent to continue to provide a lawful basis for processing irrespective of whether, in fact, the child has attained a level of maturity that would satisfy the test of *Gillick* competence in relation to the processing.

## III. A BRIGHT LINE AT AGE 16 OR 18 IN DATA PROTECTION LAW?

*Gillick* competence applies to children under age 16 but, as we have seen, in English and Welsh law, persons over age 16 but still under 18 have a statutory entitlement to consent to their own medical treatment.^41^ This raises further concerns for the data controller. In particular, how does this translate to the data protection context and using data for health research purposes? Is 16 the age at which a controller must seek consent from an individual under data protection law, or is it 18? As we have seen, the Data Protection Bill 2017 makes specific mention of the age of 16 in relation to children in Scotland, but beyond this it offers little in the way of clarity. The only other reference to children’s age is in relation to ‘information society services’. Here, the processing of personal data can only be lawful where consent has been provided by somebody over the age of 13. This does not apply to preventive or counselling services,^42^ and ‘information society services’ will typically fall outside the scope of healthcare or health-related research.

Despite legislation providing little clarity here,^43^ particularly in the context of processing data for medical purposes, it would be incongruous for an individual to be able to consent to care and treatment but not to the associated data processing. Furthermore, if a 16-year-old can consent to data processing in the context of healthcare, then why not other contexts such as health research? We argue that, particularly in light of reference to 16 years in the GDPR, and further to the caveats that we express above about the medico-legal case law dealing with the period of 16–18, there is reason to presume sufficient maturity to exercise *all* the rights of a data subject at age 16. Given this, a respect for data autonomy would suggest a responsibility on data controllers to seek an individual’s own consent when they reach age 16, including where the controller was previously reliant upon previous parental consent as the legal basis for data processing.

Although the best interest test remains relevant from age 16, as in relation to *Gillick* competence, it will be increasingly difficult to argue effectively that it is in an individual’s best interest for processing to continue if she expressly dissents. However, *if* a controller has no evidence to rebut the presumption that processing that began with a parental consent is in her best interests—and can otherwise be demonstrated to be ‘fair’ (as we discuss below)—then processing should continue after age 16 at least while efforts are made to seek her views. This position, we admit, is in opposition to the view expressed by the ICO in its draft guidance if they intended to imply 16 is the age at which a child can consent for herself. However, we suggest that the ICO guidance fails to capture sufficiently the transitory nature of a child’s growing maturity, which is precisely what *Gillick* competence attempts to do earlier in the process,^44^ and the continued application of best interests as an overriding consideration until the age of 18. Accordingly, we argue that the validity of a parental consent may still endure, but the conditions become more challenging for a data controller to meet.

The fairness of processing, while a relevant consideration to any data processing,^45^ may be considered to have special significance in circumstances where an individual independently competent to exercise a right to consent has not done so, and yet consent is relied upon as the basis for processing. Data protection law establishes a number of conditions for fair processing, including the provision of certain information relating to data processing to the data subject.^46^ We suggest that, as a minimum, to meet the requirements of fair processing, there must be at least joint communication to the child herself, as well as her parents, of the identity of the data controller and the purposes of processing (and under the GDPR a number of additional details^47^). It would need to be openly and transparently communicated to both the parents originally providing consent and the data subject the accessible means by which she can exercise her rights as a data subject. Arguably, relevant to the fairness of continued processing is also the extent to which the child was originally involved in the original decision to provide consent, and whether she assented to the processing.

We suggest that from the point that an individual might independently provide a valid consent, there should be particular efforts to provide relevant information directly to her and to establish her own view. To continue to process ignorant of her view risks her subsequently disputing any claim that processing was, in fact, in her best interests. The three conditions that need to be met, therefore, are:

1. existing parental consent extends to the ongoing processing;
2. the data controller has no reason to doubt that the processing on the basis of a parental consent will be in the best interests of a minor—at 16 or 17 years old (relevant to this last point will be any indication that the data subject dissents); and, as soon as one knows, or should presume, a child has capacity to consent on her own behalf, that
3. the continued processing on the basis of parental consent is fair.

This suggests that age 16 should *not* be considered to be a bright line at which a parental consent becomes invalid, nor a point at which a child’s consent must necessarily be secured. The fact that a data controller should assume that a child has sufficient maturity to make a decision for herself at that age only implies responsibilities to engage from that point directly with the data subject rather than her parents. In case of receiving conflicting requests from the data subject and her parents, the data controller should record reasons for resolving that conflict in the child’s best interests.

To summarise our argument so far, if a data controller comes to know, or has reason to know, that a child under 16 years of age is *Gillick* competent, then it should make reasonable efforts to ascertain the child’s view and to accommodate them, including respecting the child’s own consent. The paramount responsibility is, however, to act in the best interest of child and, therefore, to continue to process data consistent with the previous parental consent while the child’s consent is sought (assuming the processing is demonstrated to continue to be in the child’s best interest). If a child *dissents* to data processing, then the best interests’ judgement must be made taking that dissent into account. As a child approaches the age of full majority, and passes the age (16) at which competence may be presumed, it will be increasingly difficult to argue that she cannot determine for herself what is in her best interests and that processing is ‘fair’ if information about that processing has not been provided directly to her and her view sought. Arguably, it is more difficult to establish an overriding dissent to be in a child’s best interests in the context of data processing than in the context of life-saving treatment. Current guidance fails to reflect the nuance of these processes or the possible dilemmas that can arise.

## IV. FAIR AND LAWFUL PROCESSING UPON MATURITY

At the far end of the spectrum of maturity, the ICO draft guidance states that parental consent ceases to provide a valid legal basis for processing beyond the child’s attainment of legal adulthood, and in cases where the now-adult has not provided her own consent, then a legal basis other than consent would need to be established. If this is the case, then this possibility is avoided by effective engagement with the data subject prior to the age of 18. Through that contact there will have been opportunity to establish the data subject’s consent to the processing.

The fairness of continuing to process data without a valid consent, by relying upon an alternative legal basis will, at that point, be strictly scrutinised. Regulators have indicated that one must ensure it is ‘fair’ to switch from consent as the legal basis of processing to an alternative.^48^ Failure to do so may provide a data subject with an inaccurate impression of the extent to which she may exercise control, and the means by which she might exercise that control, over processing.

Therefore, if a data controller wishes to argue that it would be fair to continue to process a child’s data even after she has reached legal adulthood, then the controller would be well advised to make reasonable efforts to contact the child prior to them becoming an adult, informing them of the processing and the individual’s rights as a data subject, and provide detailed opportunities for how to consent to ongoing processing. If an alternative legal basis is available, besides consent, then the data controller should notify persons of that intention and provide clear and accessible means by which either consent may be withdrawn prior to that point or objection raised after it. Depending on the nature of the data processing operation, notice via email, letter in the post, or website notice may suffice; our questions raised at the end of Section II.C in this article, about, for example, the need for registration of research, apply here also.

## V. FAIR AND LAWFUL PROCESSING IN THE ERA OF BIG DATA

These considerations must also be set against ever-changing technical capabilities with regard to the processing of personal data. The era of Big Data is already upon us, and an early casualty will be the stubborn persistence of artificial categories—often created by law—between personal data and anonymised data, health and non-health data, research and audit, and other silos that have so far bounded much of the thinking and practice in the field of data protection. A 2017 discussion paper from the ICO reveals the extent of the challenges.^49^ The paper is an update of a 2014 document addressing the same topic, but possibilities and practices have moved on considerably in three years. For the ICO, these represent an opportunity to examine how Big Data and data protection can work together.

In this section, we briefly examine some of the emerging issues and instances of best practices as these might impact on children and young people. We suggest that it is particularly important to do so because this group of citizens is at the forefront of user uptake when it comes to emerging technological developments, yet their own emerging autonomy and legal rights are often overlooked in the discussions. As we have seen, the Data Protection Bill 2017 signals the intent to take advantage of the opportunity inherent within the GDPR to reduce the age of consent for information society services to age 13 in the UK. It is important that any apparent ‘bright line’ difference between the age of maturity for consent to use of data for medical purposes and consent to information society services is significantly obscured where individuals satisfy the *Gillick* test if that is to be a test of general application in the data protection context. In any case, the opportunities that the ICO encourage data controllers to take in the context of Big Data are directly relevant to the kinds of engagement we have described above. Our intention here, however, is not simply to bolster our claims regarding good practice in a cognate field. The reality is that guidance regarding communication and consent in the context of Big Data will be increasingly of direct significance to processing for healthcare and health research purposes.

In its 2017 paper, the ICO suggest that ‘big data analytics’ involving the linkage and sharing of potentially vast volumes of data across diverse sectors and heterogeneous settings represents a step-change in data processing and its regulation. Offering the example of education, the paper states:

Learning analytics in higher education (HE) involves the combination of ‘static data’ such as traditional student records with ‘fluid data’ such as swipe card data from entering campus buildings, using virtual learning environments (VLEs) and downloading e-resources. The analysis of this information can reveal trends that help to improve HE processes, benefiting both staff and students.^50^

When one adds to this the temporal consideration of access to earlier stage education records, the potential benefits (and risks) are further multiplied. Linking to health records might, in turn, help to reveal correlations between childhood ill health and academic performance, and all of this processing might improve future decision-making for individuals and social institutions alike. But, what of the lawfulness and legitimacy of such practices?

The ICO sees no reason why the watchwords of accountability and transparency should not drive best practice. Moreover, in legal terms, the enduring role of consent, privacy notices, privacy impact assessments, and data subject rights remain (rightly) front and centre. Of direct relevance to this article are discussions of the role of social media, and the impact of citizens’ behaviours on their expectations of privacy and the role of consent. As is self-evident, young people will often develop an early presence on social media—a ripe source of potentially valuable data for any Big Data initiative. And yet, as various surveys have pointed out, this setting can give rise to a ‘privacy paradox’:

… people may express concerns about the impact on their privacy of ‘creepy’ uses of their data, but in practice they contribute their data anyway via the online systems they use. In other words they provide the data because it is the price of using internet services.^51^

In response, the ICO points to examples of ‘graduated consent’ that is based on an ongoing relationship with a service provider, and ‘just in time’ notifications that seek citizen authorisation before a new instance of data processing takes place. Such technical measures, we contend, could also be designed-in to the regulation of relationships with children of growing maturity, perhaps having a default time trigger at the age of 16 and again at full legal capacity, but also by seeking evidence — perhaps through the medium of Big Data — of the individual child’s own developing autonomy and maturity through her online presence.^52^

In like manner, the ICO argues persuasively for imaginative uses of privacy notices that can cut through and minimise the perceived complexity of explaining Big Data, perhaps through the use of videos and cartoons, as used by enterprises such as O2 and *The Guardian*.^53^ Finally, the ICO points to the important potential role of ethical approaches to data processing in this emerging era. For example, the Information Accountability Foundation has its Big Data Ethics Initiative from which a series of ethical values have been proposed for assessing big data initiatives.^54^ These include:

- organisations should define the benefits of the analytics;
- they should not incur the risks of big data analytics if the benefits could be achieved by less risky means;
- the insights should be sustainable;
- the processing should respect the interests of stakeholders; and
- the outcomes of the processing should be fair to individuals and avoid discriminatory impacts.

Albeit developed at a high level of abstraction, similar values and proposals could be developed for initiatives that will involve children and young people, and that would include the kinds of perspectives for which we argue in this article. Thus, for example, we might include the following additional considerations:

- the benefits of the analytics must be defensible in the best interests of the children concerned;
- the design of the analytics should take account of the growing autonomy of the child, especially where the processing is on the basis of parental consent; and
- the lawful and ethical basis for the analytics should be reviewed upon maturity of the child, with clear lines of accountability and transparency.

In sum, we suggest that the era of Big Data holds considerable beneficial potential but that it also stands to impact disproportionately on younger citizens if the key messages of this article are not heeded.

## VI. ALTERNATIVES TO CONSENT AS LEGAL BASIS FOR PROCESSING

We finish by briefly reflecting on the significance of a recent move to highlight the availability of alternatives to consent as a legal basis for processing personal data. In order to be lawful under the GDPR, the processing of personal data must meet at least one of the conditions set out in Article 6(1) GDPR. Consent is but one of the available conditions for lawful processing. If processing a special category of personal data, such as data concerning an individual’s health, then it is also necessary to apply one of the conditions set out in Article 9(2) GDPR. ‘Explicit consent’ is an option here, but again only one of several alternatives.

In the draft guidance issued by the ICO on consent under the GDPR, the ICO make clear their view that the ‘GDPR sets a high standard for consent’^55^ and confirm that ‘[c]onsent is one lawful basis for processing, but there are alternatives.’^56^ In fact, they expressly guide controllers away from a reliance upon consent where they are public authorities: ‘Public authorities, employers and other organisations in a position of power over individuals should avoid relying upon consent’^57^—and the Data Protection Bill 2017 indicates that universities will be public authorities for the purposes of data protection law.^58^ Where there are appropriate alternatives to consent, then UK universities, responsible for much of the health research and the longitudinal studies we have as our focal point, are receiving a clear steer towards them and away from consent (for data protection purposes).^59^

What does this mean for the argument we have advanced? We have already considered the situation where processing starts on the basis of parental consent and then a controller considers shifting to another legal basis. What, though, if consent is not the legal basis from the outset? While the arguments in this article have application to many longitudinal studies already started, is significance of consent likely to wane as controllers shift to alternative legal bases in the future? Possibly. But we would offer two brief reflections. The first is that the relevance of the arguments put forward here remains undented for those projects that have already started on the basis of (a parental) consent. They are important projects and deserve clarity regarding a route through to lawful processing as participants mature. The second is that, while a move from consent as the legal basis for health research may occur, this is because there are alternatives to consent for health research under data protection law^60^ that are not always going to be available where data is processed for other (non-research) purposes. Here, we repeat a point made at the outset, although the arguments offered here are prompted by a concern for the ongoing legal basis for health research; as the arguments apply to the processing of children’s personal data more generally, they have a potentially broader application.

## VII. CONCLUSION

In this article, we explored the topical question of whether consent obtained from a parent for use of a child’s personal data for health research ‘lapses’ when the child is old enough to provide her own consent, and if so, when that lapse might occur, and how is it to be determined. We considered, and challenged, the suggestion in draft guidance by the ICO that parental consent will always expire when the child reaches the age at which they can consent for themselves. We argued that if the processing of a child’s data began with the consent of their parent, then there is a need to find and defend an enduring consent through the child’s growing capacity and certainly on to their maturity. The *Gillick* test can assist in determining when the shift in focus might occur from parent to child, but this does not determine any clear bright line, and it envisions a continuing role for best interest considerations. As such, it is not at all clear that the *Gillick* threshold presents an *absolute* legal requirement for a child’s consent in order for processing to be lawful, let alone a preferred ethical approach. The *Gillick* test assesses a narrow decision-specific competence. In the healthcare context, the pre-16 child’s competence to make a consent decision under the *Gillick* test exists in parallel with duties of others to act as proxy for them (with his or her best interests as an overriding consideration).

Given that best interests remain an overriding consideration until age 18, and also in the data protection context, we suggested that it is ethically preferable and legally defensible for data controllers to envision or ascertain the emerging ability of an individual to exercise the rights of a data subject first *jointly* (rather than in parallel) with a parent and then *independently* of them (rather than solely). The ability of a child to provide an independent consent does not proscribe her parents continuing to exercise a parallel duty to provide a consent where consistent with her best interests. Similarly, there is no reason to suppose a previously provided consent lapses simply due to a child achieving *Gillick* competence in relation to a specific decision. Indeed, the practical implications of such a position would likely overwhelm data controllers and discourage the processing of children where there was any possibility they may be competent in relation to a decision on processing. This too would not be in the child’s best interests.

A straightforward transplant of the *Gillick* test from the healthcare context to the realm of data protection is problematic for other reasons. The first is the requirement for ‘full understanding’ that the courts have insisted upon within the healthcare context. In Scotland, in the data protection domain, children are to be required to have only a ‘general understanding’ before they may exercise certain rights of a data subject. We have suggested that this threshold also be relied upon for data protection that applies on both sides of the border. Further, *Gillick* competence has relevance in the healthcare context up to the age of 16 (and not the age of full majority). While neither regulatory guidance nor the data protection legislation makes clear that legal competence to exercise the rights of a data subject should be presumed at the age of 16 (in England and Wales, consistent with the FLRA), we argue that the presumption *should* apply in the area of data protection, including in relation to health research. As a result, the conditions upon which a controller may rely upon a previous parental consent become more challenging. There may be limited circumstances in which a data controller considers it appropriate to continue to process data relating to a child past the age of 16, and on the basis of a parental consent. These circumstances would most likely be limited to those where the following considerations apply:

1. the requirements regarding consent were met at the time that consent was originally given;
2. there is no reason to rebut the presumption that, in line with previous parental consent, it remains in the best interest of the child that the processing continue; and
3. it would be ‘fair’ to rely upon the alternative legal basis in the circumstances.

Whether it would be ‘fair’ requires consideration of all material factors including, but not limited to,

1. whether it is clear to the data subject, ie the child, that she has rights in relation to the processing, including the right to object;
2. the extent to which the child was originally involved in the original decision to provide consent, and whether she assented to the processing;
3. the extent to which considerations of ‘best interest’ are engaged and favour processing; and
4. evidence from any context in which the child has begun to exercise her own data autonomy, for example, through social media or other means that suggest evidence of growing maturity.

If a child has been engaged appropriately with the initial decision by a parent to provide consent to processing, and subsequent communication materials have been addressed to her as well as the parent and have extended to re-enforcing the right to withdraw consent at any point, then there is at least an arguable case that a consent previously provided by a parent does not cease to provide a secure lawful basis for processing as soon as a child is old enough to provide her own consent: either as soon as she attained a particular age short of full maturity (eg 16 years), or if she could satisfy a test of *Gillick* competence in relation to the particular decision. Where they had been previously engaged as joint decision makers in giving the consent, it is reasonable to continue to presume the processing to be in her best interests. The continued processing may remain lawful *at least* while efforts to re-engage with the data subject and to seek fresh consent were made. Data controllers would be wise to develop strategies for when and how to seek fresh consent from children as soon as is practicable after the data controller has reason to be aware they are mature enough to consent on their own behalf.

We acknowledge that as one moves away from a hypothetical ideal involving full engagement and agreement between parties, it becomes more challenging to argue for fair and lawful to continue to process data for medical purposes, when original consent was provided by a parent and when this remains the sole basis for data processing. This is particularly challenging in the face of a refusal by a mature minor or someone between age 16 and 18. Post-18, where controllers have not successfully obtained consent from the child previously, then they should only normally continue to process where an alternative legal basis is available to them. Here, processing must still be fair. When assessing whether continued processing is ‘fair’, there should be an additional consideration to those listed above: whether reliance upon that alternative legal basis as a ground for processing has been openly and transparently communicated to both the parent (or other legal representative) originally providing consent and also the data subject.

In order to avoid the possibility that data controllers find themselves without a legal basis for the processing of an individual’s data after age 18 (or earlier), they would be well advised to adopt some of the techniques for effective communication now being developed in the context of Big Data. As Big Data requires a response to the challenge of relative opaque processing and dynamic purposes, so might those responses serve to engage effectively an audience that is itself dynamic and developing. The ultimate lesson here is for both productive engagement with children and to view consent as a process, just as childhood is a process towards maturity itself.

## Footnotes

1
*Gillick* competence is the test used by health professionals to determine whether a child under 16 years of age is able to consent to his or her own medical treatment, without the need for parental permission or knowledge. There is no lower age limit for *Gillick* competence to be applied. See *Gillick v West Norfolk and Wisbech Health Authority* [1986] AC 112, discussed below.

2 Information Commissioner’s Office, ‘Consultation: GDPR Consent Guidance’, 26 <https://ico.org.uk/media/about-the-ico/consultations/2013551/draft-gdpr-consent-guidance-for-consultation-201703.pdf> accessed 10 October 2017.

3 Family Law Reform Act 1969 (England and Wales); Age of Legal Capacity (Scotland) Act 1991; Age of Majority Act (Northern Ireland) 1969.

4 [1985] UKHL 7, [1986] AC 112.

5 ibid 173.

6 Care Quality Commission, ‘Gillick Competency and Fraser Guidelines’ <http://www.cqc.org.uk/content/nigels-surgery-8-gillick-competency-and-fraser-guidelines> accessed 10 October 2017. Where a court determines any question relating to a child, then s 1(1) Children Act 1989 puts the child’s welfare as the paramount consideration.

7 See s 2(4) of the Age of Legal Capacity (Scotland) Act 1991. See further, S Elliston, *Best Interests of the Child in Healthcare* (Routledge-Cavendish 2007).

8
*Re R (a Minor)(Wardship: Medical Treatment)* [1992] Fam 11.

9
*Re W (a Minor)(Medical Treatment)* [1993] Fam 64.

10 This is also the case post-16 (pre-18), in case of treatment decisions, due to the operation of s 8(3) Family Law Reform Act 1969.

11 See eg E Cave, ‘Adolescent Consent and Confidentiality in the UK’ (2009) 16 EJHL 309. Whether the asymmetry of consent and refusal available to a mature minor must be understood to be due to an underlying asymmetry of competence or instead can be explained by a notion of transitional paternalism is interestingly explored by Neil Manson in ‘Transitional Paternalism: How Shared Normative Powers Give Rise to the Asymmetry of Adolescent Consent and Refusal’ (2014) 29 Bioethics 66. See also N Manson, ‘When is a Choice Not a Choice? ‘Sham Offers’ and the Asymmetry of Adolescent Consent and Refusal’ (2017) 31 Bioethics 296.

12 V Chico and L Hagger, ‘The Mental Capacity Act 2005 and Mature Minors: A Missed Opportunity?’ (2011) 33(2) J Soc Wel Fam L 157.

13 The Latey Committee, Report of the Committee on the Age of Majority (1967) (Cmnd 3342), especially paras 474–84.

14 With the exception of the Medical Protection Society.

15
*Re W* (n 9) 78.

16 ibid 87.

17
*Re E (A Minor) (Wardship: Medical Treatment)* [1993] 1 FLR 386; [1994] 5 Med LR 73.

18
*Hewer v Bryant* [1970] 1 QB 357, 369.

19 The Article 29 Working Party is composed of representatives of the national data protection authorities in the European Union, the European Data Protection Supervisor, and the European Commission, and provides expert advice to the EU Member States regarding data protection.

20 Article 29 Data Protection Working Party, Opinion 2/2009 on the protection of children’s personal data (General Guidelines and the special case of schools) <http://ec.europa.eu/justice/policies/privacy/docs/wpdocs/2009/wp160_en.pdf> accessed 10 October 2017.

21 ibid 5.

22 ibid 6.

23 In the UK, there is statutory acknowledgement that the concept of the mature minor extends to at least some data decisions. For example, with regards to subject access requests, it has been stated that: ‘[i]n Scotland, the law presumes that a child aged 12 years or more has the capacity to make a subject access request.’ Here the test is status-based rather than functional.

24 ICO, GDPR Consent Guidance (n 2) 27.

25
*Gillick* (n 1).

26 We question, for example, the privacy and data protection implication of new technologies such as non-invasive prenatal foetal testing, which might generate whole genome sequencing data of the ‘future person’. See Nuffield Council on Bioethics, *Non-invasive Prenatal Testing: Ethical Issues* (2017), paras 3.32, 5.13, and 5.37, <http://nuffieldbioethics.org/wp-content/uploads/NIPT-ethical-issues-full-report.pdf> accessed 10 October 2017.

27 Article 29 Data Protection Working Party (n 20) 6.

28 ibid 4.

29 We acknowledge the complexities and difficulties of applying the best interests test to children as noted, inter alia, in the General Comment of the Committee on the Rights of the Child on the Child’s Best Interest (General Comment No 14 (29 May 2013)), especially relating to the risk of manipulation in assessment and implementation. For discussion, see E Cave, ‘Competence and Authority: Adolescent Treatment Refusals for Physical and Mental Health Conditions’ (2013) 8(2) Contemp Soc Sci 92.

30 This is consistent with the Nuffield Council on Bioethics’ recommendations in relation to children and clinical research that ‘children from a young age should be understood not as “subjects” of research but as “active participants”’. See Nuffield Council on Bioethics, *Children and Clinical Research: Ethical Issues* (2015) para 1.22 <http://nuffieldbioethics.org/wp-content/uploads/Children-and-clinical-research-full-report.pdf> accessed 10 October 2017. See also distinctions made within the broad concept of childhood and the responsibilities that may attach to each, in ch 4.

31 ibid 6.

32 See parallel discussion of responsibilities of parents, ibid para 4.10.

33 s 187(1)

34 s 187(2)

35 s 187(3). Note that this reflects existing provisions relating to Scotland. For example, as stated by the ICO: ‘[i]n Scotland, the law presumes that a child aged 12 years or more has the capacity to make a subject access request.’ See ICO, ‘Guide to Data Protection’ <https://ico.org.uk/for-organisations/guide-to-data-protection/principle-6-rights/subject-access-request/> accessed 10 October 2017. See also Freedom of Information (Scotland) Act 2002, s 69(2). Note also positions being taken, eg *Aberdeen City Council’s Corporate Data Protection Procedures for Third Party Request Procedures for Parents*: ‘Once a child reaches the age of 12 years they are deemed to be capable of making their own decisions with regards to their personal data.’ (para 3.1) <http://www.aberdeencity.gov.uk/nmsruntime/saveasdialog.asp?lID=59680&sID=24439> accessed 10 October 2017.

36
*Re R* (n 8) (Lord Donaldson).

37 Article 29 Data Protection Working Party (n 20).

38 See United Nations Committee on the Rights of the Children, General Comment No 14 (2013) on the right of the child to have his or her best interests taken as a primary consideration (2013) <http://www2.ohchr.org/English/bodies/crc/docs/GC/CRC_C_GC_14_ENG.pdf> accessed 10 October 2017.

39 Although, as indicated in the Children Act 1989, the parents have *rights* and duties different from, for example, a court. Parents have a duty of care to their child, but it is arguably in balance with other rights and duties in the family. Equally, they have rights to raise their child according to their sensitivities where they are not in (danger of) breach of that general duty of care.

40 Article 29 Data Protection Working Party (n 20) 5.

41 s 8, Family Law Reform Act 1969.

42 Clause 8, Data Protection Bill 2017.

43 At least not within UK data protection law or the GDPR outside the context of information society services. For discussion of consent in the context of information society services and where domestic legislation does establish a ‘bright-line’ in some EU Member States, see M Macenaite and E Kosta, ‘Consent for Processing Children’s Personal Data in the EU: Following in US Footsteps?’ (2017) 26 Inf Commun Technol L 146, esp 153, 189.

44 We discuss this further in G Laurie, ‘Liminality and the Limits of Law in Health Research Regulation: What are We Missing in the Spaces In-between?’ (2017) 25 Med L Rev 47.

45 The first data protection principle is that processing be both fair and lawful. See pt I, sch 1, Data Protection Act 1998. This principle is retained as art 5(1) GDPR.

46 See pt II, sch 1, Data Protection Act 1998.

47 Set out in art 13 GDPR.

48 See ICO, GDPR Consent Guidance (n 2) 8.

49 Information Commissioner’s Office, ‘Big Data, Artificial Intelligence, Machine Learning and Data Protection’ (June 2017) <https://ico.org.uk/media/for-organisations/documents/2013559/big-data-ai-ml-and-data-protection.pdf> accessed 10 October 2017.

50 ibid para 27.

51 ibid para 44, citing J Pybus, M Coté and T Blanke, ‘Hacking the Social Life of Big Data’ (2015) 2(2) Big Data Soc 1; SE Peacock, ‘How Web Tracking Changes User Agency in the Age of Big Data: The Used User’ (2014) 1(2) Big Data Soc 1.

52 The ICO adds the important caveat that ‘… [j]ust because people have put data onto social media without restricting access does not necessarily legitimise all further use of it.’ ibid para 64. These measures would have to be designed in such a way as to conform to the requirements of the GDPR. Recital 38 recognises that children merit special protection with regard to their personal data, in particular, eg where collecting personal data with regards to children when using services offered directly to a child. Recital 71 makes clear that children should not be the subjects of profiling *solely* on the grounds of automated processing, where there are legal or similarly significant effects. Any evidence gathered on capacity, via Big Data, might (at most) trigger human review before affecting a data controller’s understanding of a child’s capacity to consent on her own behalf.

53 ibid para 145.

54 Information Accountability Foundation, ‘Big Data Ethics Initiative’ <http://informationaccountability.org/big-data-ethics-initiative/> accessed 10 October 2017.

55 ICO, GDPR Consent Guidance (n 2) 5.

56 ibid 11.

57 ibid.

58 Clause 6, Data Protection Bill 2017.

59 See E Denham, ‘Consent is Not the ‘Silver Bullet’ for GDPR Compliance’ ICO Blog (16 August 2017) <https://iconewsblog.org.uk/2017/08/16/consent-is-not-the-silver-bullet-for-gdpr-compliance/> accessed 10 October 2017. It is worth recognising that consent will continue to be an important means by which conformity with other legal requirements—such as those associated with the common law duty of confidence—are met.

60 Universities are likely to be able to rely upon art 6(1)(e) GDPR where processing personal data for the purposes of research and art 9(2)(j) where processing a special category of personal data (including data concerning health) for research purposes, as long as the relevant conditions are met.

